# Comparison of five bacterial strains producing siderophores with ability to chelate iron under alkaline conditions

**DOI:** 10.1186/s13568-019-0796-3

**Published:** 2019-05-28

**Authors:** Carlos M. H. Ferreira, Ângela Vilas-Boas, Cátia A. Sousa, Helena M. V. M. Soares, Eduardo V. Soares

**Affiliations:** 1 REQUIMTE/LAQV, Departamento de Engenharia Química, Faculdade de Engenharia, Universidade do Porto, rua Dr. Roberto Frias, 4200-465 Porto, Portugal; 2 Bioengineering Laboratory-CIETI, Chemical Engineering Department, ISEP-School of Engineering of Polytechnic Institute of Porto, rua Dr António Bernardino de Almeida, 431, 4249-015 Porto, Portugal; 3 CEB-Centre of Biological Engineering, University of Minho, 4710-057 Braga, Portugal

**Keywords:** Catechol and hydroxamates-type siderophores, Environment-friendly chelating agents, Iron chelation under alkaline conditions, Microorganisms, Siderophore production

## Abstract

Iron deficiency is one of the main causes of chlorosis in plants, which leads to losses in field crops quality and yield. The use of synthetic chelates to prevent or correct iron-deficiency is not satisfactory mainly due to their poor biodegradability. The present work aimed to search suitable microorganisms to produce alternative, environment-friendly iron-chelating agents (siderophores). For this purpose, the performance of five bacteria (*Azotobacter vinelandii*, *Bacillus megaterium*, *Bacillus subtilis*, *Pantoea allii* and *Rhizobium radiobacter*) was evaluated, regarding siderophore production kinetics, level of siderophore production (determined by chrome azurol S, CAS method), type of siderophore produced (using Arnow and Csaky’s tests) and iron-chelating capacity at pH 9.0. All bacteria were in stationary phase at 24 h, except *A. vinelandii* (at 72 h) and produced the maximum siderophore amount (80–140 µmol L^−1^) between 24 and 48 h, with the exception of *A. vinelandii* (at 72 h). The analysis of culture filtrates revealed the presence of catechol-type siderophores for *B. subtilis* and *R. radiobacter* and hydroxamate-type siderophores for *B. megaterium* and *P. allii*. In the case of *A. vinelandii*, both siderophore-types (catechol and hydroxamates) were detected. The highest iron-chelating capacity, at pH 9.0, was obtained by *B. megaterium* followed by *B. subtilis* and *A. vinelandii.* Therefore, these three bacteria strains are the most promising bacteria for siderophore production and chlorosis correction under alkaline conditions.

**Electronic supplementary material:**

The online version of this article (10.1186/s13568-019-0796-3) contains supplementary material, which is available to authorized users.

## Introduction

Iron is an essential nutrient for plant development together with macronutrients (Barker and Stratton 2015). Although not present in chlorophyll, iron is necessary for its synthesis and for the functioning of the photosynthetic apparatus (Broadley et al. 2012). The lack of iron in plants leads to develop a symptomatic array, named chlorosis. It is characterized by the development of yellow young leaves as a result of insufficient iron for the efficient chlorophyll production (Lucena 2000). Chlorotic plants will under develop, produce less biomass and yield less flowers and fruits, or, in ultimate case, lead to complete crop failure (Guerinot and Yi 1994). Some crops known to be susceptible to iron chlorosis are peach, kiwifruit, citrus and pear (Lucena 2003), although some other valuable crops have also been described to suffer from chlorosis, such as rice (Yoshida et al. 1996), soybean (Froechlich and Fehr 1981), tomato (Chaney et al. 2008) and pea (Kabir et al. 2016).

The low bioavailability of iron is due to its chemical nature and its low solubility and dissolution kinetics, which limits the absorption of iron by plants, especially in calcareous soils. Since it is estimated that calcareous soils cover about 30% of world´s cultivated soils (Barker and Stratton 2015; Cianzio et al. 2006), this data underlines the importance of iron deficiency as a major global agricultural problem. To surpass the problem of chlorosis, organic chelating agents have been used, such as aminopolycarboxylic acids (APCAs), to correct iron deficiency. Although, these compounds are strong chelating agents, usually they are not biodegradable. Thus, their persistence and consequent accumulation in aquatic systems is becoming a matter of great concern, as they can mobilize toxic metals (Bucheli-Witschel and Egli 2001). Therefore, the search for environmental-friendly and suitable iron chelators, with specific properties to be used, as iron fertilizers, has become a great challenge. Among a great number of compounds considered, siderophores are an interesting object of study since they are effective iron chelating compounds and have the advantage of being more biodegradable than synthetic APCAs (Fazary et al. 2016a, b).

Siderophores are low weight molecules (between 500 and 1500 dalton) with great affinity and selectivity to bind and complex Fe(III). They are produced by microorganisms, as well as by some gramineous plant, as part of a strategy to obtain iron from the environment because of the low amount of iron bioavailable (Hider and Kong 2010). This particular trait has drawn the attention of many researchers in recent years. For instance, some studies have been conducted in search on new siderophore producing species. Grobelak and Hiller (2017) have conducted a screening of bacteria (*Bacillus* spp. and *Pseudomonas* spp.) isolated from plant’s roots for catechol and hydroxamate producing bacteria. The positive effects of siderophore producing bacteria have been recently studied by different authors (Liu et al. 2017; Sabaté et al. 2017; Trifi et al. 2017).

Considering that iron deficiency is a yield-limiting factor with major implications for crop management, the production of siderophores compounds is an important challenge. In recent works (Ferreira et al. 2019a; Martins et al. 2018), it was shown that synthetic compounds containing catecholate and hydroxamate groups are potential iron chelators for iron nutrition in plants. However, due to the high structural complexity of siderophores, its production by chemical synthesis involves several low yield steps, which limits the feasibility of their use for agriculture purposes, as it is illustrated in the synthesis of azotochelin (Leydier et al. 2008; Martins et al. 2018). One alternative is the microbial (biotechnological) production of siderophores.

The present work aimed to seek for bacteria that can be further used in the production of environment-friendly suitable iron chelators. These compounds should be produced at low cost, in order to be used in agriculture as iron fertilizers. For this purpose, five bacterial strains of different genera were chosen taking into account the following criteria: (i) ability to produce catecholates or hydroxamates siderophores, as these compounds display the highest affinity to iron (Hider and Kong 2010); (ii) absence of pathogenicity to humans, i.e., belonging to risk 1 (U.S. Department of Health and Human Services et al. 2009). These strains were evaluated in order to select those which seem to be the most promising siderophore producers, considering the following parameters: efficiency of iron-complexing capacity at pH 9.0, amount of siderophore produced, type of siderophore, speed of siderophore production, growth and handling of the strain (growth, nutritional requirements, culture medium composition and easiness of biomass separation from the culture medium).

## Materials and methods

### Microorganisms, media and culture conditions

As a result of a literature survey previously performed for siderophore producer bacterial strains (Ferreira et al. 2019b) and taking into to account the selection criteria presented above, the following microorganisms were chosen to be used in the present work: *Azotobacter vinelandii* Deutsche Sammlung von Mikroorganismen und Zellkulturen (DSM) 2289; *Bacillus megaterium* American Type Culture Collection (ATCC) 19213; *Bacillus subtilis* DSM 10; *Pantoea allii* DSM 25133; and *Rhizobium radiobacter* DSM 30205. The original strains were obtained from DSM, Germany, or ATCC, U.S.A. All these bacteria belong to Risk 1 group, according to the U.S. Department of Health and Human Services, Centers for Disease Control, and National Institutes of Health (2009).

The microorganisms were maintained in minimal medium (MM) agar, except *A. vinelandii,* which was maintained in Burk´s medium (BM) agar, at 4 °C. MM agar contained per litre: 10 g glucose, 1.47 g glutamic acid, 3.0 g potassium hydrogenophosphate (K_2_HPO_4_), 1.0 g potassium dihydrogenophosphate (KH_2_PO_4_), 0.5 g ammonium chloride (NH_4_Cl), 0.1 g ammonium nitrate (NH_4_NO_3_), 0.1 g sodium sulphate (Na_2_SO_4_), 10 mg magnesium sulphate heptahydrate (MgSO_4_·7H_2_O), 1 mg magnesium sulphate tetrahydrate (MnSO_4_·4H_2_O), 0.5 mg calcium chloride (CaCl_2_) and 20 g agar. BM agar was prepared as previously described (HiMedia Laboratories 2015) replacing sucrose by glucose; the medium contained per litre: 10 g glucose, 0.8 g K_2_HPO_4_, 0.2 g KH_2_PO_4_, 0.20 g MgSO_4_·7H_2_O, 0.253 mg sodium molybdate, 0.13 g calcium sulphate and 20 g agar. The final pH of the media was set to 7.0 ± 0.1. For iron-replete media, 29 mg of iron(III) chloride (FeCl_3_) was also added.

All reagents used were obtained from Merck (Darmstadt, Germany), Panreac (Barcelona, Spain), Sigma-Aldrich (St. Louis, Missouri, EUA) or BD Difco (Waltham, Massachusetts, EUA). Inorganic chemicals were pro-analysis grade while organic chemicals were Ph. Eur. grade.

In order to avoid iron contamination (on iron-deficient-cultures), all glassware was soaked in 10% nitric acid, overnight and, subsequently, washed with deionized water prior to use.

All pre-starter cultures, with exception of *A. vinelandii,* were prepared by inoculating the bacteria in 20 mL of iron-replete MM broth in 100 mL Erlenmeyer flasks. Cells were incubated at 30 °C, for 8 h, in an orbital shaker at 150 rpm. Starter cultures were prepared by inoculating 40 mL of iron-replete MM broth, in 100 mL Erlenmeyer flasks, with an appropriate volume of the pre-starter cultures, and then incubated overnight to an OD_600_ ≈ 2.0, under the same conditions described for the pre-starter cultures. Cells, in exponential phase of growth, were harvested by centrifugation (3000×*g*, 10 to 30 min), rinsed and suspended in iron-deficient media. Next, cultures were obtained by inoculating the cells in 400 mL of iron-deficient MM broth (initial OD_600_ ≈ 0.1), in 1 L Erlenmeyer flasks. Cells were incubated under the same conditions as described above. For *A. vinelandii*, there were some differences in the protocol: BM broth was used instead of MM broth and only a starter culture was grown, for 24 h, due to the slower growth rate of the bacterium.

### Determination of siderophore production

Due to its easiness and ability for a high and sensitive detection of siderophores (Schwyn and Neilands 1987), the chrome azurol S (CAS) method was used (Alexander and Zuberer 1991) in the estimation of the bacterial siderophore production. Therefore, samples were taken and cells were pelleted by centrifugation (3000×*g*, 10 to 30 min). The supernatant was carefully removed and, subsequently, filtered through a 0.45 μm pore size filter and immediately stored at − 20 °C until siderophore determination.

CAS method consists in mixing 1.0 mL of filtrate, properly diluted with deionized water, with 1 mL of CAS assay solution, prepared as described by Alexander and Zuberer (1991). One reference solution was also prepared by mixing 1.0 mL of CAS solution with 1.0 mL of deionized water. A zero-absorbance solution was also prepared by mixing 1.0 mL of CAS solution with 1.0 mL of 100 µmol L^−1^ desferrioxamine mesylate salt (desferal). The solutions were left to reach chemical equilibrium at room temperature in the dark for 24 h. Next, absorbance was read at 630 nm. A calibration curve was performed by plotting the ratio A/A_ref_ versus the concentration of desferal; where, A is the standard solution absorbance at 630 nm and A_ref_ stands for absorbance of the reference at 630 nm. Siderophore production is expressed as µmol L^−1^ desferal equivalent. For each culture, samples were read in triplicate and repeated independently at least two times (that is, two independent cultures read in triplicate).

### Siderophore qualification

The type of siderophore present in each bacterial medium was characterized using Arnow’s and the Csaky’s methods for catecholates and hydroxamates, respectively (Payne 1994).

Arnow method is based on the reaction between catechol and nitrite-molybdate reagent, in acidic conditions, originating a yellow colour. The colour changes to an intense orange-red in alkaline conditions. For this purpose, 1.0 mL of culture filtrate was combined with 1.0 mL of HCl 0.5 mol L^−1^. Subsequently, 1.0 mL of nitrite-molybdate reagent was added and then 1.0 mL of NaOH 1.0 mol L^−1^. The assay was incubated at room temperature, for approximately 5 min, to allow full colour development. As blank, 1.0 mL of deionised water was used. Nitrite-molybdate reagent was prepared by dissolving 10 g of sodium nitrite and 10 g of sodium molybdate in 100 mL of deionized water. If catecholate-type siderophore is present, the solution presents an orange-red colour. The colour intensity is dependent of the amount of catechol present (Arnow 1937).

The Csaky’s test detects hydroxamate-type siderophores and depends on oxidation to nitrite and formation of a coloured complex via diazonium coupling (Csáky 1948). Firstly, 1.0 mL of culture filtrate was hydrolysed with 1.0 mL of 6 N H_2_SO_4_, at 130 °C, for 30 min. The solution was then buffered with the aid of 3.0 mL of sodium acetate (350 g L^−1^) and 1.0 mL of sulfanilic acid [10 g L^−1^, 30% acetic acid (v/v)] was added, followed by 0.5 mL of iodine solution (13 g L^−1^, in glacial acetic acid). The solution was allowed to settle for 3–5 min, after which the excess of iodine was neutralized by the addition of 1.0 mL of 20 g L^−1^ sodium arsenite. Finally, 1.0 mL of α-naphthylamine solution [3 g L^−1^, in 30% (v/v) acetic acid] was added and the colour was allowed to develop for 20–30 min. The presence of hydroxamates in solution was confirmed by the presence of a deep pink colour.

Synthetic *N*,*N*-dihydroxy-*N*,*N*′-diisopropylhexanediamide (DPH), a hydroxamate, and Azotochelin, a catecholate, were used as controls (Martins et al. 2018). As blank, 1.0 mL of deionised water was used in both assays.

### Complexation capacity assays

The complex capability of each culture medium containing siderophore was tested using a procedure adapted from Villen et al. (2007). Briefly, to a fixed volume of culture filtrate, FeCl_3_ was added, the solution pH was set to 9.0 ± 0.1 and let to rest for 30 min. Then, pH was corrected again and let to settle for 3 h. The solution was subsequently centrifuged (3000×*g*, 10 min) and filtered by a 0.45 µm pore size membrane. The amount of Fe in solution was then determined (see “Iron determination” bellow). A graphical representation of the ratio [Fe]_complex_/[L] versus [Fe]_added_/[L] was plotted in order to represent the iron complexation capability of the siderophore in solution; where [Fe]_complex_ is the concentration of Fe found in the filtrate, [Fe]_added_ is the total Fe concentration added and [L] is the concentration of siderophore determined by CAS method.

### Bacteria staining

Bacterial cultures were collected and cells were fixed with 3.5% (w/v) formaldehyde for 2 h. Cells were then harvested by centrifugation and re-suspended in 10 mmol L^−1^ phosphate-buffered saline (PBS) solution (pH 7.0) with 3.5% (w/v) formaldehyde and stored at 4 °C, until to be observed.

Previously stored cells were pelleted by centrifugation and washed twice with PBS buffer (pH 7.0). Cells were re-suspended in PBS buffer with 3 µmol L^−1^ 4,6-diamidino-2-phenylindole (DAPI) and incubated for 15 min in the dark at room temperature. DAPI is a cell membrane permeant stain, which exhibits a strong blue fluorescence upon bonded to adenine–thymine rich regions in DNA (Haugland 2005).

### Microscopy

Bacteria were observed by phase-contrast or epifluorescence using a microscope with a HBO 100 mercury lamp and the A filter (excitation filter BP 340–380, dichromatic mirror 400, and suppression filter LP 425) from Leica. The images were captured with a Leica DC 300F camera (Leica Microsystems, Heerbrugg, Switzerland) using a 100 × oil immersion N plan objective and processed using Leica IM 50-Image manager software.

### Iron determination

Iron determinations in cell cultures and in complexation capacity assays were carried out by atomic absorption spectroscopy with flame atomization (AAS-FA) using a Perkin Elmer AAnalyst 400 spectrometer (Norwalk, CT, USA).

### Reproducibility of the results

All experiments were repeated, independently, at least, two times. Data is presented as mean values. Growth-curves were performed in duplicate; for each time, growth was monitored in triplicate. Data reported for siderophore concentrations and iron complexation experiments are the mean ± standard deviations of at least six determinations.

## Results

### Microbial growth and siderophore production

**Fig. 1:**
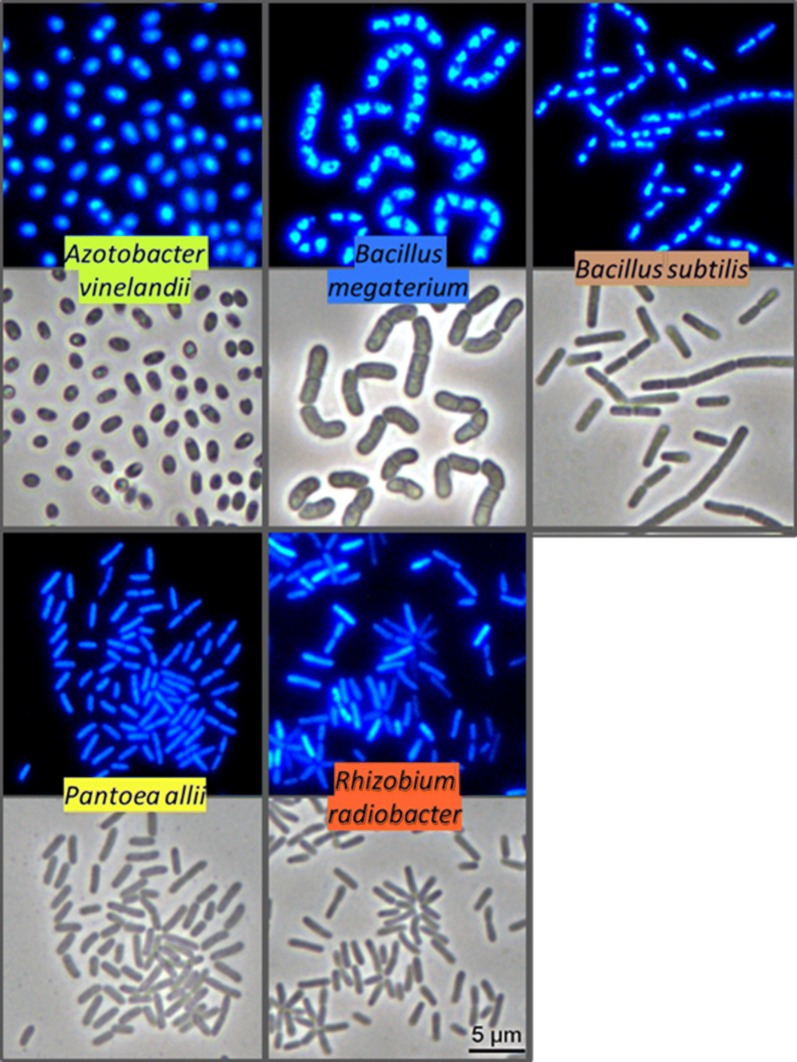
Visualization of the morphology of the bacteria studied. Microphotographs illustrative of the bacteria stained with DAPI and observed by fluorescent microscopy (upper images) or by phase-contrast microscopy (lower images). All microphotographs were taken in cultures with cells in exponential phase of growth: 6 h for all strains, except *A. vinelandii* (48 h)

In the present work, it was our objective to select promissory siderophore producing bacteria that can be used in the agriculture in the correction of chlorosis, in calcareous soils. For this purpose, five bacteria (Fig. 1) were selected to cover different genus and siderophores types.

All tested bacteria were grown at 30 °C, with agitation (150 rpm), in MM broth, except *A. vinelandii* which was grown in BM broth. The pre-cultures were prepared in iron-complete media. To limit iron carryover (from iron-complete media), which inhibit siderophore production, cells in exponential phase of growth were washed and re-inoculated in the same media but without iron. At the end of the growth, the iron concentration in culture media was below the detection limit (0.09 mg L^−1^) of the method used.

**Fig. 2:**
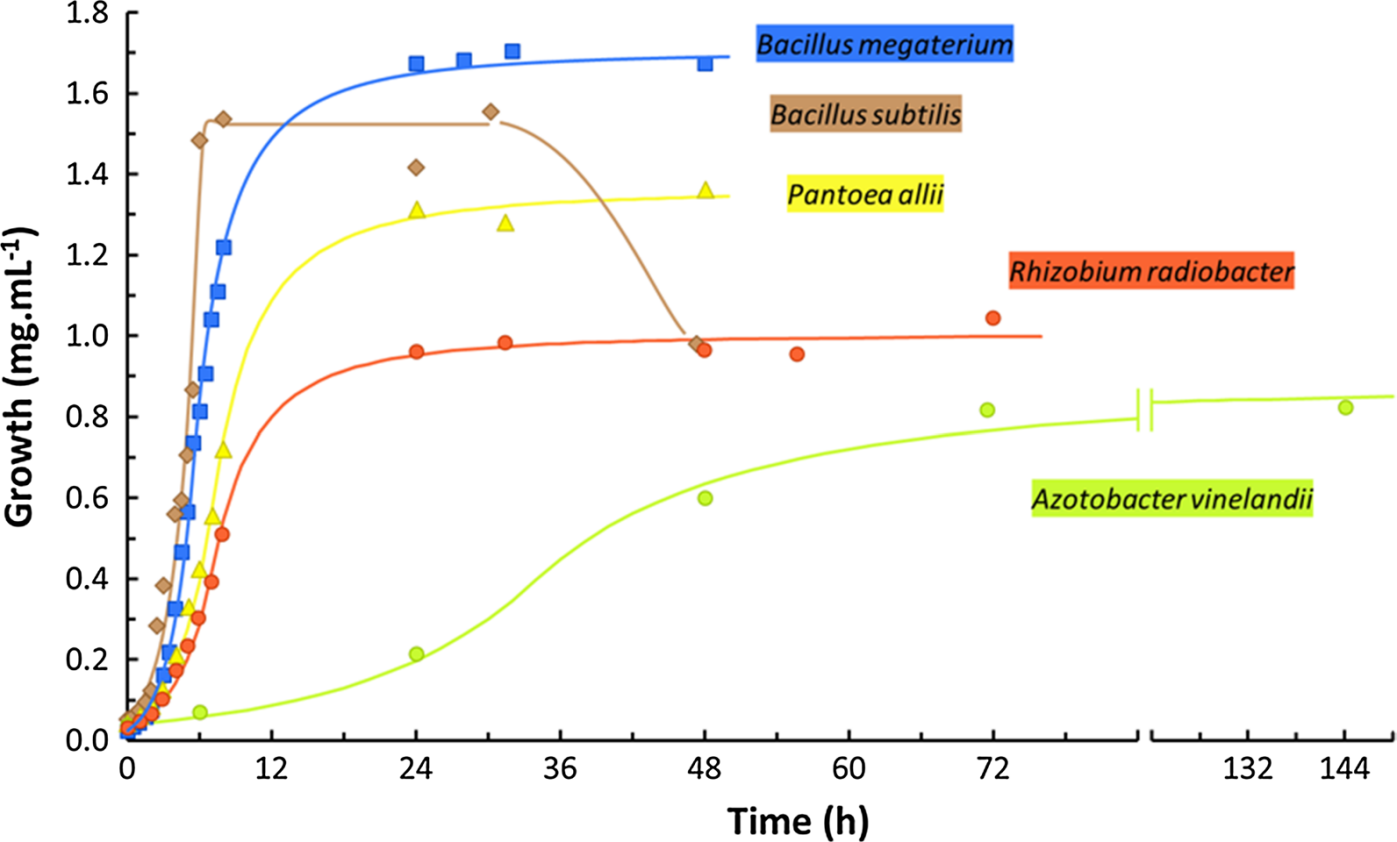
Growth of the bacteria studied. The bacteria, in exponential phase of growth, were inoculated in minimal medium (MM) broth (except *A. vinelandii*, which was inoculated in Burk´s medium broth), iron-deficient, and incubated at 30 °C with agitation (150 rpm). This is a typical example of an experiment performed at least two times. Each point represents the average of three determinations

With the exception of *A. vinelandii*, all bacteria grown in MM had a duplication time between 1.1 and 1.8 h (Additional file 1: Table S1) and were in stationary phase of growth at 24 h of incubation (Fig. 2). *A. vinelandii*, a bacterium with the ability to fix atmospheric nitrogen (Hamilton et al. 2011), grew more slowly in BM broth (a culture medium without nitrogen source). Under the cultural conditions used, *A. vinelandii* presented a duplication time of about 14.4 h (Additional file 1: Table S1) and was in stationary phase at 72 h (Fig. 2).

The amount of biomass reached, in stationary phase, varied widely. After 24 h of growth, *B. megaterium* and *B. subtilis* reached a biomass of 1.7 and 1.5 mg mL^−1^, respectively, while *R. radiobacter* only reached a biomass of ~ 1 mg mL^−1^ (Fig. 2). *A. vinelandii* had a maximum biomass less than 1 mg mL^−1^ while *P. allii* had a maximum biomass of 1.3 mg mL^−1^ (Fig. 2). In the case of *B. subtilis,* after 30 h of growth, a decrease of biomass was observed (Fig. 2), probably due to the formation of spores (Additional file 1: Figure S1). A similar behaviour was reported for other *Bacillus* species (Bharucha et al. 2013; Ait Kaki et al. 2013; Santos et al. 2014).

**Fig. 3:**
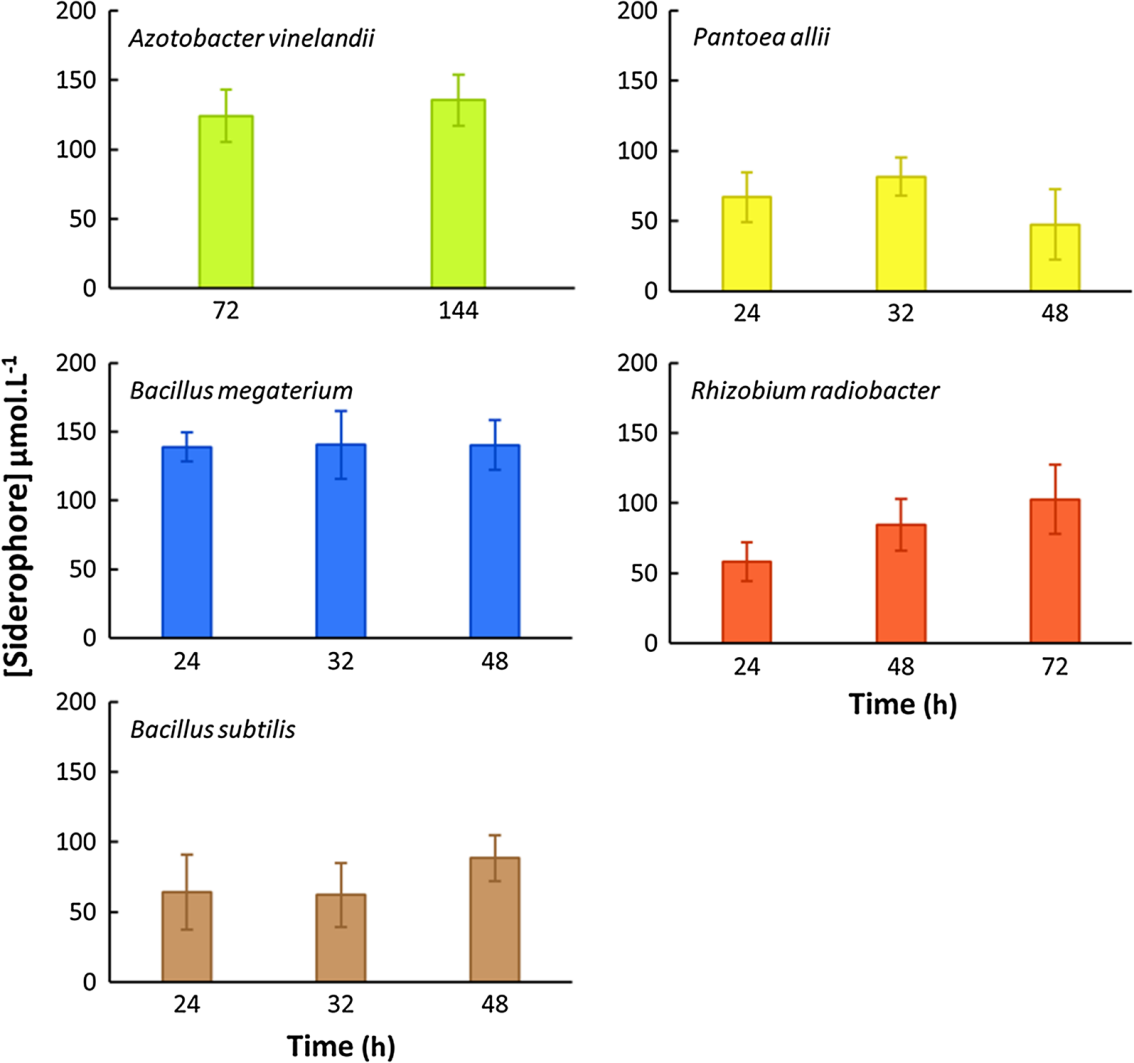
Siderophore production by the different bacteria tested. Siderophore was quantified by CAS assay on culture filtrates and expressed as µmol L^−1^ desferal equivalent. Each bar represents the mean (± SD) of at least six determinations

Siderophore production was followed and compared, over time, using the CAS method (Alexander and Zuberer 1991). Under the cultural conditions tested (culture medium composition, temperature and agitation), the siderophore concentration found in the culture filtrates of *B. megaterium* reached the maximum level at 24 h (Fig. 3), when the bacterium was in stationary phase (Fig. 2). After this time, the level of siderophore remained stable (Fig. 3). Although delayed, a similar pattern of siderophore production was observed for *A. vinelandii.* Siderophore concentration in *B. subtilis* and *P. allii* filtrates were also relatively stable from 24 to 48 h of growth; only a small increase or decrease of the mean values were observed at 48 h, respectively (Fig. 3). In the case of *R. radiobacter*, an increase of the mean value of siderophore production was observed between 24 and 48 h (Fig. 3).

### Siderophore qualification

The type of siderophore present in each medium filtrate was tested using the Arnow’s and Csaky’s tests. Arnow’s test allows the identification of the presence of catechol groups in siderophores, while Csaky’s test is used to identify the presence of hydroxamic groups (Arnow 1937; Csáky 1948).

*Azotobacter vinelandii* had a positive result for both Arnow’s and Csaky’s test, which indicates the presence of both catechol and hydroxamate siderophores in the filtrate of the culture medium (Table 1; Additional file 1: Figure S3). The two *Bacillus* species studied produce different siderophore types. *B. megaterium* had a strong reaction to Csaky’s and none to Arnow’s, which indicate the presence of a hydroxamate-type siderophore in the supernatant of the culture. *B. subtilis* had a strong positive reaction with the Arnow’s test and no reaction in Csaky’s, which suggest the presence of catechol-type siderophore (Table 1; Additional file 1: Figure S3). *R. radiobacter* filtrate also presented a positive result for Arnow’s test, which indicates that this microorganism produces a catechol-type siderophore. *P. allii* had a positive result on Csaky’s test, which suggests the presence of hydroxamate-type siderophores in the filtrate (Table 1; Additional file 1: Figure S3).

**Table 1:** Characterization of the siderophore-type produced by the bacteria

Bacteria	Arnow^a^	Csaky^a^
*Azotobacter vinelandii*	+	+
*Bacillus megaterium*	−	++
*Bacillus subtilis*	++	−
*Pantoea allii*	−	+
*Rhizobium radiobacter*	+	−

^a^Catechol- and hydroxamate-type siderophores were identified using Arnow´ and Csaky’s tests, respectively. ++ strong positive result; + positive result; − negative result

### Iron complexation capacity

The problem of plant chlorosis is particularly critical under alkaline conditions due to the low concentration of iron bioavailable (Hansen et al. 2006; Colombo et al. 2014). Bacterial filtrates can be further used in the preparation of siderophore concentrate intended to be used in the chlorosis amendment in calcareous soils. In order to assess the iron chelating potential of the culture filtrates, for the pH range typical of calcareous agronomic conditions (pH 9.0), the bacterial culture filtrates were subjected to the procedure described in “Materials and methods”, “Complexation capacity assays”.

**Fig. 4:**
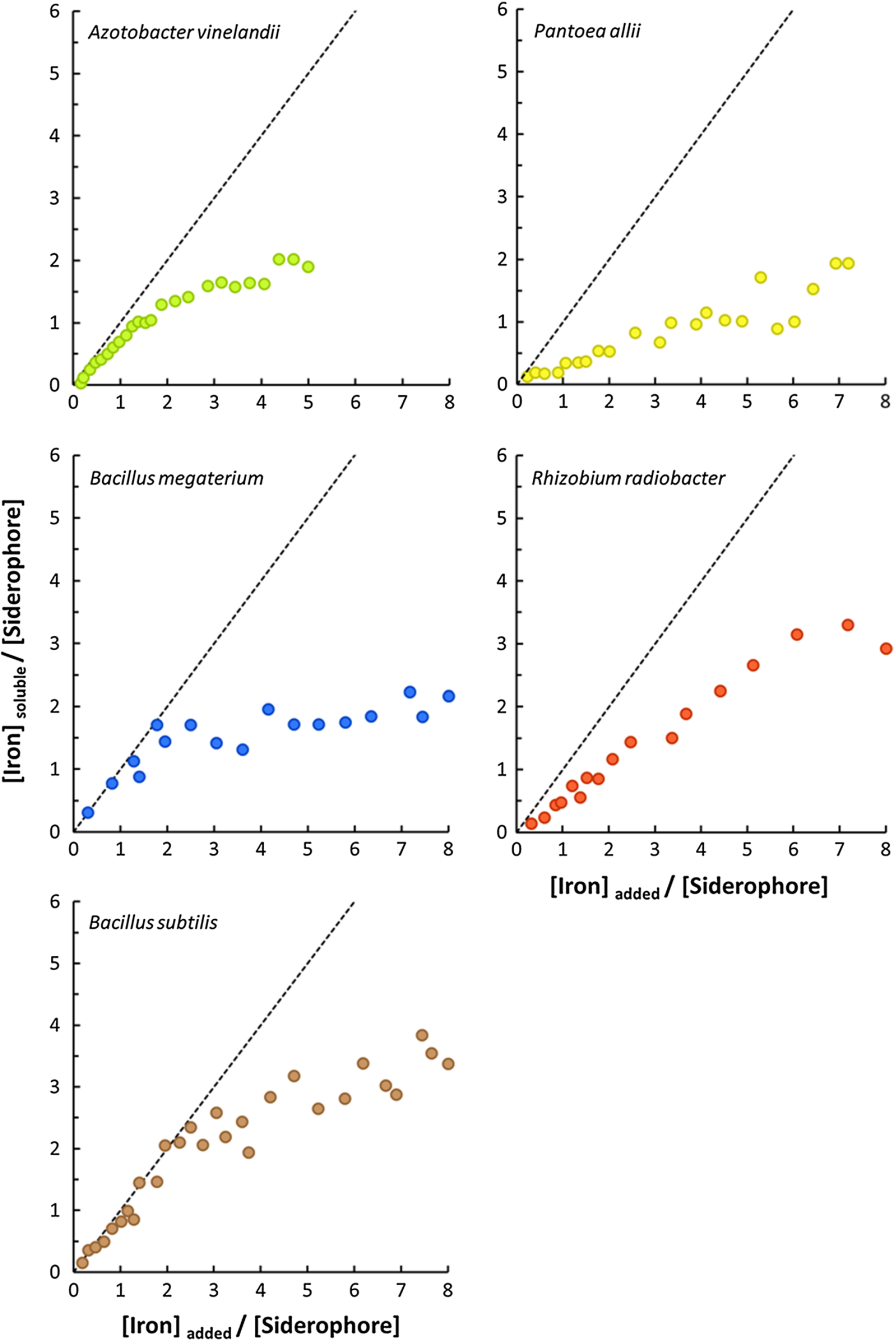
Average of complexed iron versus added iron as function of total siderophore concentration as determined by CAS method. Dashed grey line represents a line with slope = 1

The main results of the complexation capacity assays are shown in Fig. 4. In regard to *B. megaterium* and *B. subtilis*, the initial slopes of the experimental data are close to 1 (dashed line) up to a $\left[ {Fe} \right]_{added} /\left[ {Siderophore} \right]$ ratio of about 2 and 2.5, respectively, which indicates that iron is being complexed with fairly good effectiveness up to that amount of iron added. For *A. vinelandii*, although some early deviations are seen, the only major shift from the slope = 1 is seen at $\left[ {Fe} \right]_{added} /\left[ {Siderophore} \right]$ ratio of about 1.5. At this point, an efficiency of about 75% is observed with $\left[ {Fe} \right]_{added} /\left[ {Siderophore} \right]$ of 1.5 corresponding to a $\left[ {Fe} \right]_{soluble} /\left[ {Siderophore} \right]$ of 1. In both cases, early deviations may be related to inefficient complexation in the pH range used (pH 9.0 ± 0.1); on the other hand, more severe deviations observed for higher $\left[ {Fe} \right]_{added} /\left[ {Siderophore} \right]$ ratios are more likely due to the complexation of weaker siderophores/chelating agents, where the added iron is not effectively complexed and promptly precipitates.

For each culture filtrate, based on the values of highest $\left[ {Fe} \right]_{added} /\left[ {Siderophore} \right]$, one can, based upon CAS values, estimate the amount of Fe efficiently complexed. For example, in the case of *A. vinelandii* filtrate, considering a CAS reading of 140 µmol L^−1^, one can expect to have 140 µmol L^−1^ of dissolved iron upon addition of about 210 µmol L^−1^ of Fe (1.5 × 140 µmol L^−1^). Likewise, a *B. subtilis* filtrate, with a CAS reading of 100 µmol L^−1^, is expected to be able to dissolve the entirety of 250 µmol L^−1^ of added Fe (2.5 × 100 µmol L^−1^).

On the other hand, *P. allii* and *R. radiobacter* evidenced lower slopes from early on, which is somehow contradictory to what would be expected. For example, literature describes that *R. radiobacter* produces agrobactin, which complex iron in a 1:1 ratio, in an hexadentate conformation; thus, a slope close to 1 would be expected (Fig. 4) as a result of the high complex stability and consequent effectiveness of complexation. The observed results may be due to the lower efficiency for chelating iron at this pH and/or due to the presence of other(s) siderophore(s) with a different stoichiometry (and stability). Due to the lack of information of the siderophores produced by *P. allii* and the results shown in Fig. 4, it is possible that the siderophores produced are not of the 1:1 type, as it is the case of enterobactin, desferrioxamine, and aerobactin, which were described for others species of *Pantoea* (Soutar and Stavrinides 2018). Nonetheless, in case of a 1:1 complexation, the complexed formed may not be very strong and, thus, not capable of complexing most iron added at pH 9.0.

## Discussion

### Definition of the culture time for siderophore production

It was our aim to select microorganisms producers of efficient siderophores, such as catecholates and hydroxamates, since these compounds display the highest affinity to iron (Hider and Kong 2010). A search in the literature revealed the presence of more than 20 bacteria genera that produces these siderophore-types (Bhattacharyya and Jha 2012; Ferreira et al. 2019b; Hider and Kong 2010). From these, only bacterial strains non-pathogenic to humans (risk 1 group) (U.S. Department of Health and Human Sevices et al. 2009) were considered as adequate for this work.

In a general way, the maximum level of siderophore in cultures filtrates was attained when the bacteria entered the stationary phase (Figs. 2 and 3). The amount of siderophore produced remained approximately constant after 24 h for *B. megaterium*, *B. subtilis and P. allii* or after 72 h for *A. vinelandii*. Only for *R. radiobacter,* a small increase of siderophore production was observed in the beginning of the stationary phase (24–48 h) (Figs. 2 and 3). Unless for *A. vinelandii*, these results indicate that, for future siderophore production using these bacteria, it is not necessary to prolong the culture more than 48 h; for *A. vinelandii*, it is necessary to prepare a culture with 72 h.

The pattern of growth and the siderophore production of *B. subtilis* strain studied are in agreement with those described in the literature (Patel et al. 2009). However, under the cultural conditions used in the present work, the strain presented a higher level of siderophore compared to that described by Miethke et al. (2006) using a minimal culture medium. Also, a faster maximum concentration of siderophore production was achieved with our conditions than those used by Fazary et al. (2016a) to grow *Bacillus* spp. ST13. In the present work, for the bacterium *B. megaterium*, the maximum concentration of siderophore was found when the cultured reached the stationary phase (Figs. 2 and 3). Using a different culture medium and different cultural conditions, it was described that *B. megaterium* continued to produce siderophore during the stationary phase (Santos et al. 2014). A similar pattern was here observed, between 24 and 48 h, with *R. radiobacter*. Using a different *Rhizobium* species (*R. militoli* and a *Rhizobium* strain, isolated from cowpea), a maximum siderophore production was described after 24 h of growth (Jadhav and Desai 1992; Reigh 1991).

### Selection of the best iron complexation performance under alkaline conditions

As reported above, strains producers of catecholate and/or hydroxamate-type siderophores are desirable due to the high iron binding capacity of these compounds. Therefore, to confirm the presence of such compounds in the bacterial filtrates, the respective siderophore qualification was carried out.

Several siderophores have been described for *A. vinelandii*, namely azotobactin, previously known as yellow-green fluorescent peptide (Bulen and LeComte 1962), 2,3-dihydroxybenzoic acid, azotochelin (Corbin and Bulen 1969), aminochelin (Page and Tigerstrom 1988) and protochelin (Cornish and Page 1995), which are, respectively, mono-, mono-, di-, mono- and tri-catecholates. Vibrioferrin, another siderophore described (McRose et al. 2017), has two hydroxy-carboxylate moieties which should not react in Csaky and Arnow tests. Finally, azotobactin has a complex structure in which a catechol and a hydroxamate moieties can be found (Duhme et al. 1998). The positive result obtained in both tests (Table 1), can be attributed to the possible presence of azotobactin, aminochelin, azotochelin or/and protochelin. Given the less intense colour developed in the case of *Azotobacter* culture compared to the one observed in the case of *B. subtilis* (Additional file 1: Figure S3), a lower quantity of catecholate may be expected. Considering the similar average molar quantities of siderophores on *B. subtilis* and *A. vinelandii* filtrates evaluated by CAS (~ 100 µmol L^−1^ vs ~ 120 µmol L^−1^) and the more intense colour developed by *B. subtilis* compared to *A. vinelandii*, these facts suggest that the number of catechol sites per molecule may be lower in the case of *A. vinelandii* filtrates. Therefore, the presence of mono and di catecholates, such as azotobactin, aminochelin and/or azotochelin is very likely. Given the positive result for Csaky’s test and the fluorescent hue of the filtrate (Additional file 1: Figure S2), the presence of azotobactin is very plausible. The positive result in Csaky’s test for *B. megaterium* is in agreement with the literature, which describes the production of schizokinen and its derivatives by this bacterium, under iron-limited conditions (Mullis et al. 1971). Schizokinen is a di-hydroxamate siderophore with a citrate backbone (Goldman et al. 1983; Hu and Boyer 1995). On the other hand, the positive result in Arnow’s test for *B. subtilis* is compatible with the literature, which describes the ability of this bacterium to produces bacillibactin (a tri-catecholate siderophore, which has a 1:1 coordination with iron) and, to a less extent, the more simple itoic acid, with a coordination of 3:1 (Dertz et al. 2006). *R. radiobacter* also presented a positive result in Arnow’s test; this result is in agreement with the literature, which describes the ability of *R. radiobacter* to produce agrobactin, a tri-catecholate with a structure simpler than the one produced by *B. subtilis* (Ong et al. 1979). For *P. allii*, little information regarding siderophore production and/or structure has been published. Although it was described that a strain similar to *P. allii* had great siderophore production capacity, no information regarding the type of siderophore was provided (Pereira and Castro 2014). For other Pantoea species, a recent genome-wide survey has found three gene clusters homologous to those of enterobactin (catecholate-type siderophore), desferrioxamine (hydroxamate-type siderophore), and aerobactin (hydroxamate-type siderophore) (Soutar and Stavrinides 2018). These findings are in agreement with the results found for *P. allii* as a hydroxamate-type siderophore producer (Csaky positive test).

Even though all bacteria tested in this work evidenced siderophore production of similar order of magnitude (Fig. 3) (when evaluated by the CAS assay and expressed as µmol L^−1^ desferal equivalent), these results do not mean that these culture filtrates have ability to complex Fe at high pH conditions, which represent the real situation that the complexes have to endure when they are applied in alkaline soils.

The siderophores present in the culture filtrates of *A. vinelandii, B. megaterium and B. subtilis* evidenced high stability to complex Fe at pH 9.0 in a wide range of $\left[ {Fe} \right]_{added} /\left[ {Siderophore} \right]$ (up to 1.5, 2.0 and 2.5, respectively) whereas the other two cultures (*P. allii* and *R. radiobacter*) revealed weaker ability (Fig. 4). *R. radiobacter* produces agrobactin, which complex iron in a 1:1 ratio, in a hexadentate conformation; thus, a slope close to 1 would be expected (Fig. 4) as a result of the high complex stability and consequent effectiveness of complexation. In this work, the weaker complexation capacity may be due to the lower efficiency for chelating iron at this pH and/or due to the presence of other(s) siderophore(s) with a different stoichiometry (and stability). Due to the lack of information of the siderophores produced by *P. allii* and the results shown in Fig. 4, it is possible that the siderophores produced are not of the 1:1 type, as it is the case of enterobactin, desferrioxamine, and aerobactin, which were described for others species of *Pantoea* (Soutar and Stavrinides 2018). Nonetheless, in case of a 1:1 complexation, the complexed formed may not be very strong and, thus, not capable of complexing most iron added at pH 9.0.

### Overall comparison of bacteria performance

In order to select the strain(s) producer(s) of siderophores with potential ability to be subsequently tested for correcting iron chlorosis in plants, the performance of the bacteria was compared regarding the following characteristics: iron complexation capacity at pH 9.0; siderophore type; growth and handling of the strain: growth rate, nutritional requirements (culture media composition) and easiness of biomass separation from the culture medium (Table 2).

**Table 2:** Summary of the main properties of the bacteria studied

Bacteria	Average complexation capacity at pH 9.0 (µmol L^−1^)	Siderophore type^a^	Growth and handling^b^
*Azotobacter vinelandii*	188	C+H	+++
*Bacillus megaterium*	280	H	+++
*Bacillus subtilis*	225	C	+
*Pantoea allii*	Weak	H	+
*Rhizobium radiobacter*	Weak	C	+

^a^Siderophore type: C = catecholate; H = hydroxamate

^b^Growth and handling: it was considered the growth rate of the bacteria, culture media composition and easiness of the bacterial removal by centrifugation/filtration

*Azotobacter vinelandii*, *B. megaterium* and *B. subtilis* revealed to be the bacteria with the best performance since the respective medium filtrate had the ability to complex iron efficiently at pH 9.0 with the highest iron complexation capacity (Table 2). This is an indispensable condition for the culture filtrates to be used in the preparation of a siderophore concentrate intended for chlorosis amendment in calcareous soils. Also, the culture media used for bacterial growth are cheap since they are only composed by mineral salts and glucose. In the case of BM broth (used for *A. vinelandii*), the medium is selective due to the lack of nitrogen source, which reduces the risk of culture contamination during the biotechnological production of the siderophore. The low growth rate of *A. vinelandii* (Additional file 1: Table S1) is not a hindrance to the industrial use of this bacterium since the maximum concentration is achieved within 72 h, which is a culture time in the same order of magnitude of the other bacteria studied. Probably, due to its size (Fig. 1) and clump formation, biomass separation of *A. vinelandii* and *B. megaterium* by centrifugation/filtration is easy to perform. However, in the case of *B. megaterium*, sporulation should be avoided as spores will make filtration more difficult. To avoid this problem, the bacteria should not be left to grow more than 48 h (Fig. 2). Biomass separation of *B. subtilis,* by centrifugation/filtration, presented some difficulties, which can be attributed to the relatively small size of the bacterium (Fig. 1). It also suffers from sporulation issues as *B. megaterium.*

The siderophores produced by *P. allii* and *R. radiobacter,* presented inefficient iron-complexation capacity at pH 9.0 (Table 2), which prevent their use for agricultural purposes. *P. allii* suffers from the same handling issues reported for *B. subtilis*. Probably, due to their small size (Fig. 1), these bacteria easily clog the pores, being hard to filter.

In conclusion, in the present study, the performance of five bacteria was studied in view of their potential use for iron-induced chlorosis amendment in calcareous soils. *A. vinelandii*, *B. megaterium and B. subtilis* were the most promising, taking into account their strong iron complexation capacity at pH 9.0 (this is an important parameter if correction of iron-induced chlorosis in plants grown in calcareous soils is the aim) and the type of siderophore produced (catechol and hydroxamate). The three bacteria also presented the maximum siderophore production, evaluated by CAS assay, between 24 and 72 h, did not require expensive ingredients in culture media formulation and are easily removed (except *B. subtilis*) from the culture medium by centrifugation/filtration.

## Additional file

**Additional file 1: Figure S1.** Microphotographs illustrative of the morphology of *B. subtilis* in stationary phase of growth (culture at 48 h). **Figure S2.** Representative photographs of microbial filtrate at different times of growth. **Figure S3.** Qualification of the siderophores produced by the bacteria. **Table S1.** Duplication time and growth media for the bacteria studied.

## Data Availability

All data analysed throughout this study are shown in this article. All strains and reagents were purchased in microbial collections or in companies, respectively, specified in the text.
