# Supplementary material for: Comparison of five bacterial strains producing siderophores with ability to chelate iron under alkaline conditions
Source: AMB Express. 2019 May 28;9:78. doi: 10.1186/s13568-019-0796-3 (PMC6538730; doi:10.1186/s13568-019-0796-3)
Supplement: Supplementary file 1 — Additional file 1: Figure S1. Microphotographs illustrative of the morphology of B. subtilis in stationary phase of growth (culture at 48 h). Figure S2. Representative photographs of microbial filtrate at different times of growth. Figure S3. Qualification of the siderophores produced by the bacteria. Table S1. Duplication time and growth media for the bacteria studied. [file 13568_2019_796_MOESM1_ESM.pdf]

AMB Express

Additional file 1

Comparison of five bacterial strains producing siderophores with ability to chelate iron under alkaline conditions

Carlos M. H. Ferreira[1,2,3], Ângela Vilas-Boas[1], Cátia A. Sousa[1,2,3], Helena M. V. M. Soares[1], Eduardo V. Soares[2,3*]*

[1]REQUIMTE/LAQV, Departamento de Engenharia Química, Faculdade de Engenharia, Universidade do Porto, rua Dr. Roberto Frias, 4200-465 Porto, Portugal

[2]Bioengineering Laboratory-CIETI, Chemical Engineering Department, ISEP-School of Engineering of Polytechnic Institute of Porto, Rua Dr António Bernardino de Almeida, 431, 4249-015 Porto, Portugal

[3]CEB-Centre of Biological Engineering, University of Minho, 4710-057 Braga, Portugal

CONTENTS

Figure S1. Microphotographs illustrative of the morphology of *B. subtilis* in stationary phase of growth (culture at 48 h)……………………………………….......... S2
Figure S2. Representative photographs of microbial filtrate at different times of growth ………………………………………………..………………………. S3
Figure S3. Qualification of the siderophores produced by the bacteria ……………………….. S4
Table S1. Duplication time and growth media for the bacteria studied ……………………….. S5

[*]**Corresponding authors:**

E-mail addresses: hsoares@fe.up.pt (Helena M.V.M. Soares); evs@isep.ipp.pt (Eduardo V. Soares).

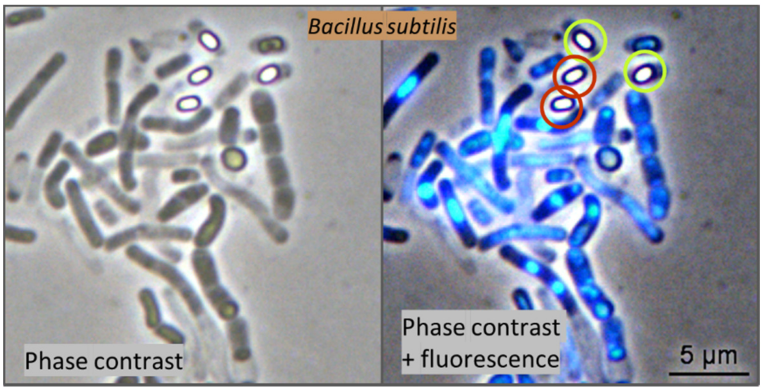

Figure S1. Microphotographs illustrative of the morphology of *B. subtilis* in stationary phase of growth (culture at 48 h). The bacterium was stained with DAPI as described in material and methods. Red circles: free spores; green circles: endospores.

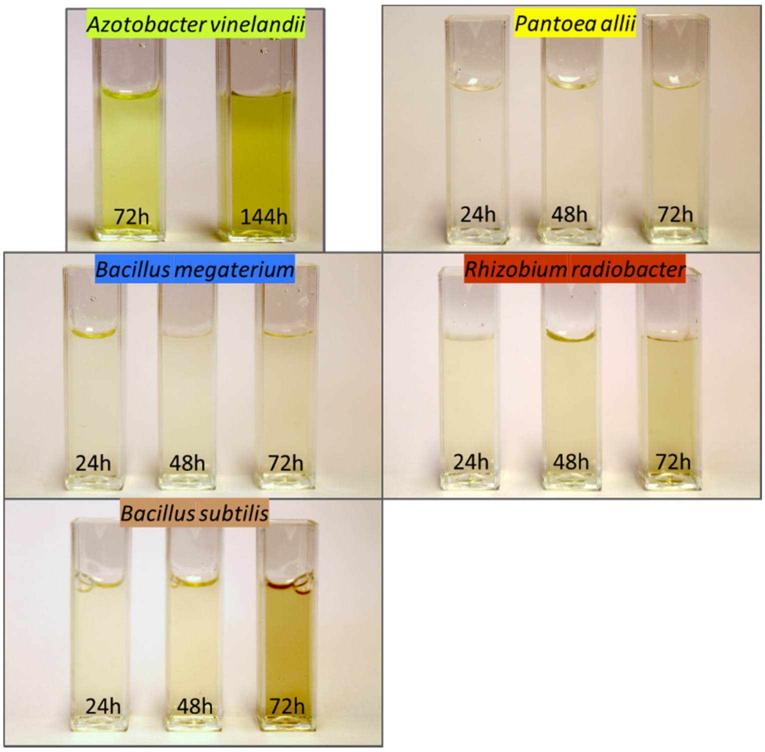

Figure S2. Representative photographs of microbial filtrate at different times of growth.

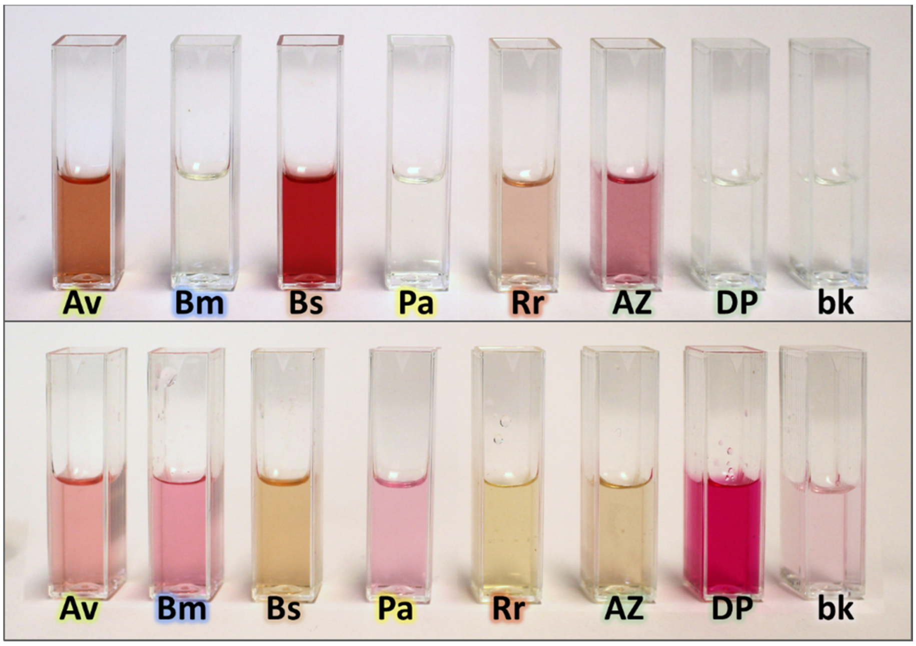

Figure S3. Qualification of the siderophores produced by the bacteria. Arnow (top) and Csaky's (bottom) tests conducted in culture filtrates. Av: *Azotobacter vinelandii*; Bm: *Bacillus megaterium*; Bs: *Bacillus subtilis*; Pa: *Pantoea allii*; Rr: *Rhizobium radiobacter*; AZ: Azotochelin, positive control for catecholates; DP: DPH, positive control for hydroxamates; Bk: blank.

Table S1. Duplication time and growth media for the bacteria studied.

Bacteria	Duplication time (h)	Growth media
Azotobacter vinelandii	14.4	Burk’s medium
Bacillus megaterium	1.1	Minimal medium
Bacillus subtilis	1.2	Minimal medium
Pantoea allii	1.7	Minimal medium
Rhizobium radiobacter	1.8	Minimal medium
